# Expression and Localization of Cathepsins B, D and G in Cancer Stem Cells in Liver Metastasis From Colon Adenocarcinoma

**DOI:** 10.3389/fsurg.2018.00040

**Published:** 2018-06-07

**Authors:** Shreeja Mehrotra, Susrutha K. Wickremesekera, Helen D. Brasch, Bede Van Schaijik, Reginald W. Marsh, Swee T. Tan, Tinte Itinteang

**Affiliations:** ^1^Gillies McIndoe Research Institute, Wellington, New Zealand; ^2^Department of General Surgery, Upper Gastrointestinal, Hepatobiliary & Pancreatic Section, Wellington Regional Hospital, Wellington, New Zealand; ^3^University of Auckland, Auckland, New Zealand; ^4^Wellington Regional Plastic, Maxillofacial and Burns Unit, Hutt Hospital, Wellington, New Zealand

**Keywords:** colorectal cancer, cathepsin, cancer stem cells, liver, metastasis, renin-angiotensin system, bypass loops

## Abstract

**Aim:**

We have previously identified and characterized cancer stem cell (CSC) subpopulations in liver metastasis from colon adenocarcinoma (LMCA). In this study we investigated the expression and localization of cathepsins B, D and G, in relation to these CSCs.

**Methods:**

3,3-Diaminobenzidine (DAB) immunohistochemical (IHC) staining for cathepsins B, D and G was performed on 4μm-thick formalin-fixed paraffin-embedded LMCA sections from nine patients. Immunofluorescence (IF) IHC staining was performed on three representative samples of LMCA from the original cohort of nine patients, to determine the localization of these cathepsins in relation to the CSC subpopulations. NanoString mRNA analysis and Western Blotting (WB) were used to examine the transcript and protein expression of these cathepsins, respectively. Enzyme activity assays were utilized to determine their functional activity. Data acquired from counting of cells staining positively of the cathepsins on the DAB IHC-stained slides and from Nanostring mRNA analysis were subjected to statistical analyses to determine significance.

**Results:**

DAB IHC staining demonstrated expression of cathepsins B, D and G within LMCA. IF IHC staining demonstrated the expression of both cathepsin B and cathepsin D by the OCT4^−^ cells within the tumor nests and the OCT4^+^ CSC subpopulation within the peritumoral stroma. NanoString mRNA analysis showed significantly greater transcript expression of cathepsin B and cathepsin D, compared to cathepsin G. WB confirmed expression of cathepsin B and cathepsin D proteins, while cathepsin G was below detectable levels. Enzyme activity assays showed functional activity of cathepsin B and cathepsin D.

**Conclusion:**

Our study demonstrated novel finding of the expression of cathepsin B, cathepsin D, and possibly cathepsin G by the putative CSC subpopulations within LMCA.

## Introduction

Colorectal cancer (CRC) is the third most common cancer worldwide, and the second major cause of cancer-related deaths in developed countries (1). In New Zealand, there are approximately 2,800 new cases of CRC annually with approximately a 50% mortality resulting from the disease (2).

The etiology and pathogenesis of CRC is complex (3) with environmental and lifestyle factors playing a significant role in colorectal carcinogenesis. An increase in the incidence of CRC paralleling economic development and adoption of a Western lifestyle, has been reported in several countries (4). Dietary and lifestyle risk factors include a high intake of unsaturated fats and red meat, total energy intake, excessive alcohol use and sedentary lifestyle (3). Somatic and inherited mutations have also been implicated (3).

Over 90% of CRC are adenocarcinoma (5). The liver is the most frequent site of CRC metastasis, via the portal venous system (1). Liver metastasis is a significant contributor to mortality in patients with CRC, with 60–70% of patients who die from advanced CRC demonstrating liver metastasis (6). Over half of patients with CRC will develop liver metastasis during their lifetime. In patients with metastatic CRC, survival is seldom more than three years despite advancements in chemotherapy and biological agents. Liver resection provides the greatest likelihood of long-term survival in these cases, with five-year survival rates of up to 58%. However, only around 20% of cases are resectable at the time of diagnosis (7, 8).

The cancer stem cell (CSC) concept of cancer proposes that a small subset of cells within the tumor - the CSCs, possess innate biological characteristics for self-renewal (9). CSCs are thought to be responsible for tumorigenesis, tumor differentiation, maintenance, spread and relapse (9, 10). Recent reports have demonstrated the expression pattern of specific CSC markers, such as CD133, CD44, leucine-rich repeat-containing G protein-coupled receptor 5 (LGR5) and epithelial cell adhesion molecule (EpCAM), in CRC (11). There is also evidence indicating the presence of CSC subpopulations within CRC, with unique expression patterns of embryonic stem cell (ESC) markers (11).

We have previously identified and characterized three subpopulations of CSCs within liver metastasis from colon adenocarcinoma (LMCA): a SOX2^+^/NANOG^+^/KLF4^+^/c-Myc^+^/OCT4^+^ subpopulation and a SOX2^+^/NANOG^+^/KLF4^+^/c-Myc^+^/OCT4^−^ subpopulation confined to the peritumoral stroma (PTS), and a SOX2^+^/NANOG^+^/KLF4^+^/c-Myc^+^/OCT4^−^ subpopulation within the tumor nests (TNs) (12). We speculate the existence of a hierarchy of CSCs within LMCA, with the OCT4^+^ cells within the PTS putatively representing the most primitive CSC subpopulation (12).

The renin-angiotensin system (RAS) is known classically for its role in regulating blood pressure and fluid and electrolyte balance. There is increasing evidence indicating dysregulation of the RAS in malignancy and is associated with poor patient outcomes (13). The paracrine mechanisms of local RAS may affect tissue angiogenesis, cellular proliferation, apoptosis and inflammation, thereby playing an important role in tumorigenesis (14). Inhibition of the RAS restrains tumor growth, metastasis and angiogenesis (13). Components of the RAS are expressed by CSC subpopulations in a number of cancer types including isocitrate dehydrogenase-wildtype glioblastoma (IDHWGB) (15) and oral cavity squamous cell carcinoma (OCSCC) affecting different subsites (16–18). These CSCs have been proposed as a potential therapeutic target by manipulation of the RAS. Cathepsins B, D and G are proteases, which provide bypass loops for the RAS (19) are also expressed by GB (20) and oral tongue SCC (21), suggesting potential treatment targeting these cathepsins.

Cathepsin B, a cysteine protease, converts inactive pro-renin into active renin (20, 22). Cathepsin D, an aspartyl protease, functionally similar to renin, converts angiotensinogen to angiotensin I (20, 23). Cathepsin G, a serine protease, produces angiotensin II from angiotensin I and directly from angiotensinogen (20, 24).

Cathepsins are produced as inactive proenzymes that undergo processing to become active enzymes. Cathepsin B is initially generated as an *N*-glycosylated proenzyme of 39 kDa and is subsequently modified under acidic conditions to the single-chain form of mature enzyme of 29 kDa (25). This process is strongly inhibited by pepstatin, an aspartic protease inhibitor, suggesting that an aspartic protease plays a role in propeptide-processing of the proenzyme (25). Cathepsin D is synthesized as a preproenzyme comprised of 412 amino acid residues (26). Following removal of the signal peptide within the endoplasmic reticulum, the proezyme is transported to an acidic compartment, where it is subsequently activated by proteolytic removal of a 44-amino acid residue prodomain. The mature form of cathepsin D can exist as either a single or two-chain form, with both forms displaying equal activity (26). Cathepsin G is a serine protease of the chymotrypsin family. It undergoes proteolytic activation by cathepsin C and is stored within neutrophil azurophilic granules as an active protease (27, 28).

Cathepsins have been implicated in invasion and metastasis in CRC, due to their ability to degrade extracellular matrix (ECM) in response to inflammatory and oncogenic stimuli (6, 29).

In this study, we investigated the expression and localization of cathepsins B, D, and G in relation to the CSC subpopulations within LMCA we have previously identified (12) using immunohistochemical (IHC) staining, immunofluorescence (IF) IHC staining, Western blotting (WB), and NanoString mRNA expression analysis. Enzymatic activity assays were performed to determine functional activity of these cathepsins. The presence of these cathepsins may underscore potential treatment target in the treatment of LMCA.

## Methods

### Tissue Samples

The same LMCA samples from nine male patients, aged 50–80 (mean, 65) years (Table 1) included in our previous study (12), were sourced from the Gillies McIndoe Research Institute Tissue Bank for this study which was approved by the Central Regional Health and Disability Ethics Committee (ref. no. 15/CEN/106). Written informed consent was obtained from all patients.

**Table 1:** Characteristics of Liver Metastasis from Colon Adenocarcinoma Samples.

Sample Number	Age (Years)	Sex	Liver Segment
1	80	M	8
2	66	M	5
3	51	M	6
4	75	M	1
5	70	M	8
6	50	M	8
7	67	M	7
8	69	M	8
9	53	M	5

### Histochemical and Immunohistochemical Staining

Hematoxylin and eosin (H&E) staining was performed on 4μm-thick formalin-fixed paraffin-embedded LMCA sections from nine patients to confirm the presence and areas of TNs and the PTS, by an anatomical pathologist (HDB). These LMCA sections then underwent DAB IHC staining, as previously described (12), using primary antibodies for cathepsin B (1:1,000; cat# sc-6490-R, Santa Cruz, CA, USA), cathepsin D (1:200; cat# NCL-CDm, Leica, Newcastle-upon-Tyne, UK), cathepsin G (1:200; cat# sc-33206, Santa Cruz, CA, USA), EMA (ready-to-use, cat# PA0035, Leica), and OCT4 (1:30, cat# MRQ-10, Cell Marque, Rocklin, CA, USA). All DAB IHC-stained slides were mounted in Surgipath Micromount (Leica).

IF IHC staining was performed to determine co-expression of two proteins on three samples of LMCA from the original cohort of nine patients used for DAB IHC staining. Vectafluor Excel anti-mouse 488 (ready-to-use; cat# VEDK2488, Vector Laboratories, Burlingame, CA, USA) and Alexa Fluor anti-rabbit 594 (1:500; cat# A21207, Life Technologies, Carlsbad, CA, USA) were utilized to detect the combinations. All IF IHC-stained slides were mounted in Vecta Shield Hardset mounting medium with 4′,6′-diamino-2-phenylindole (Vector Laboratories).

Positive control tissues used for the primary antibodies were human placenta for cathepsin B; human breast carcinoma for cathepsin D; mouse bone marrow for cathepsin G; and human seminoma for OCT4, as previously described (21). A negative LMCA control sample was prepared for DAB IHC staining by using an IgG isotype control (ready-to-use; cat# IR600, Dako, Santa Clara, CA, USA). For IF IHC staining, a negative control was performed using a section of LMCA tissue with the combined use of primary isotype mouse (ready-to-use; cat# IR750, Dako, Copenhagen, Denmark) and rabbit (read-to-use; cat# IR600, Dako) antibodies. All antibodies were diluted with Bond primary antibody diluent (cat# AR9352, Leica), and DAB and IF IHC staining was carried out on the Leica Bond Rx autostainer, as previously described (12).

### Image Analysis

DAB IHC-stained slides were viewed using an Olympus BX53 microscope and photographed with an Olympus DP21 digital camera (Olympus, Tokyo, Japan). IF IHC-stained slides were viewed and imaged using an Olympus FV1200 biological confocal laser-scanning microscope and processed with cellSens Dimension 1.11 software using 2D deconvolution algorithm (Olympus).

### Cell Counting and Statistical Analyses

Using the Olympus BX53 light microscope fitted with an Olympus DP21 digital camera, cell counting was performed using ImageJ (https://imagej.net/Welcome) on six fields of view of each DAB IHC-stained slide of the nine LMCA samples at 400x magnification. Fields of view were selected from regions that exhibited the highest density of staining. The proportion of cells within the TNs and the PTS stained positively in each field of view was calculated and the results were subjected to *t*-tests for related samples, using SPSS v.22 statistical package.

### NanoString mRNA Expression Analysis

RNA extracted from six snap-frozen LMCA samples from the same cohort of nine patients used for DAB IHC staining, was analyzed using NanoString nCounter™ Gene Expression Assay (NanoString Technologies, Seattle, WA, USA), as previously described (12). Probes for the genes encoding for cathepsin B (NM_001908.2), cathepsin D (NM_001909.3), cathepsin G (NM_001911.2), and the housekeeping gene GUSB (NM_000181.1) were used in the analysis. Raw data was analyzed by nSolverTM software (NanoString Technologies) using standard settings and results were normalized against the housekeeping gene, GUSB, and subjected to *t*-tests for related samples, using SPSS v.22 statistical package, to determine the relative abundance of each cathepsin to each other.

### Western Blotting

Total protein was extracted and precipitated from three snap-frozen LMCA samples of the original cohort of nine patients included for DAB IHC staining, separated by SDS-PAGE and transferred to a PVDF membrane using previously described methods (30). Detection of the proteins was performed on the iBind Flex (cat# SLF2000, Thermo Fisher Scientific) using the primary antibodies cathepsin B (1:250; cat# SC-6490-R, Santa Cruz), cathepsin D (1:250; cat# SC-6486, Santa Cruz), cathepsin G (1:250; cat# ab197354, Abcam, Cambridge, UK), and β-actin (1:500; cat# ab8226, Abcam). Appropriate secondary antibodies were goat anti-rabbit Alexa Fluor 647 (1:2000; cat# A21244, Life Technologies) for cathepsin B, chicken anti-goat Alexa Fluor 647 (1:2000; cat# A21469, Life Technologies) for cathepsin D, goat anti-mouse Alexa Fluor 488 (1:2000; cat# A21202, Life Technologies) for β-actin, and goat anti-rabbit HRP (1:2000; cat# ab6721, Abcam) for cathepsin G. Clarity Western ECL (cat# 1705061, Bio-Rad) was used as the substrate for visualizing HRP detected protein bands and the Chemi Doc MP Imaging System (Bio-Rad) and Image Lab 5.0 software (Bio-Rad) were used for band detection and analysis. All experiments were performed in triplicates. Snap-frozen tonsillar tissue was used as control tissue for cathepsin B and cathepsin D with a recombinant cathepsin G protein (cat# H00001511-Q01, Novus Biologicals, Littleton, CO, USA) as the control for cathepsin G. Matched mouse (1:500; cat# ab18443, Abcam) and rabbit (1:500; cat# ab171870, Abcam) isotype controls were used as appropriate negative controls.

### Enzymatic Activity Assays

Enzymatic activities of cathepsin B and cathepsin D were determined in snap-frozen LMCA samples from the same three patients used in WB, using enzymatic activity assay kits for cathepsin B (cat# ab65300; Abcam) and cathepsin D (cat# ab65302; Abcam), as previously described (21). Fluorescence was measured in a Nunc^TM^ F96 MicroWell^TM^ black polystyrene plate (cat# 136101, Thermo Fisher Scientific) using the Varioskan Flash plate reader (cat# MIB5250030, Thermo Fisher Scientific). Tonsil and denatured tonsil tissue lysates were used as appropriate positive and negative controls, respectively. All experiments were performed in duplicates with averages taken.

## Results

### Histochemical and DAB IHC Staining

**Figure 1:**
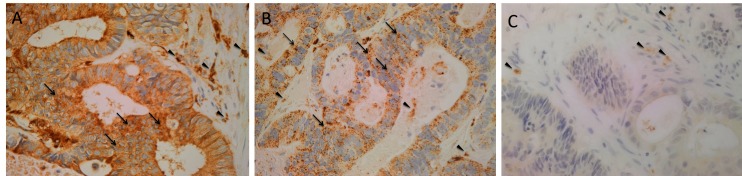
Representative 3,3-diaminobenzidine immunohistochemical-stained sections of liver metastasis from colon adenocarcinoma demonstrating cytoplasmic expression of cathepsin B [**(A)**, brown] within the tumor nests (TNs, *arrows*) and the peritumoral stroma (PTS, *arrowheads*). Granular cytoplasmic staining of cathepsin D [**(B)**, brown] was localized to cells within the TNs (*arrows*) and those within the PTS. Cytoplasmic expression of cathepsin G [**(C)**, brown] was demonstrated in cells within the PTS (*arrowheads*). Nuclei were counter-stained with hematoxylin [**(A-C)**, blue]. Original magnification: 400×.

H&E staining of 4 μm-thick formalin-fixed paraffin-embedded sections of all nine LMCA samples confirmed the presence of the tumor on the slide by an anatomical pathologist (HDB). DAB IHC staining showed cytoplasmic expression of cathepsin B (Figure 1A, brown) by cells predominantly within the TNs (*arrows*) and some cells within the PTS (*arrowheads*). Granular cytoplasmic staining of cathepsin D (Figure 1B, brown) was also localized to cells within the TNs (*arrows*) and some cells within the PTS (*arrowheads*). Cytoplasmic expression of cathepsin G (Figure 1C, brown) was localized to a few cells within the PTS (*arrowheads*). Interestingly there was minimal staining for cathepsin B (Figure S1A, brown), cathepsin D (Figure S1B, brown) and cathepsin G (Figure S1C, brown) on the adjacent normal hepatocytes.

Positive controls for cathepsin B (Figure S1D, brown), cathepsin D (Figure S1E, brown) and cathepsin G (Figure S1F, brown) demonstrated the expected staining patterns in human placenta, breast carcinoma and mouse bone marrow, respectively. The negative control demonstrated no staining (Figure S1G).

### IF IHC Staining

**Figure 2:**
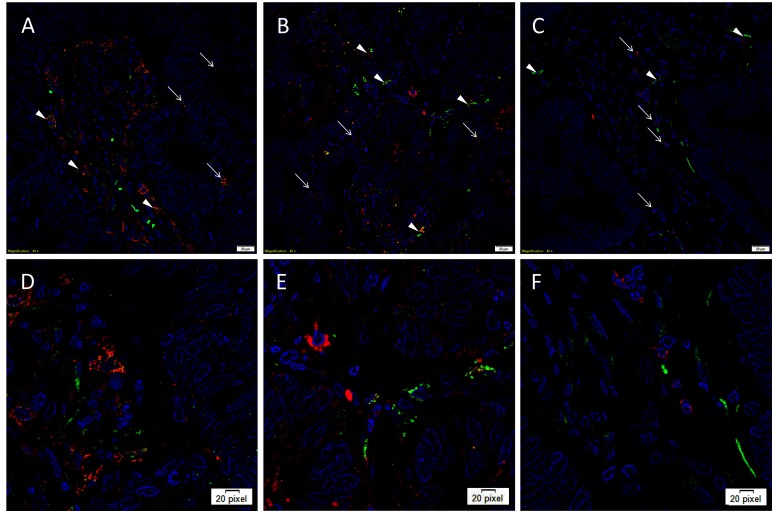
Representative immunofluorescence immunohistochemical-stained sections of liver metastasis from colon adenocarcinoma demonstrating expression of cathepsin B [**(A, D)**, red] by cells within the TNs (*arrows*) and the OCT4^+^ cells within the peritumoral stroma [**(A, D)**, green, *arrowheads*]. Cathepsin D [**(B, E)**, red] was similarly expressed by cells within the TNs (*arrows*) and the OCT4^+^ CSC subpopulation [**(B, E)**, green, *arrowheads*]. Cathepsin G [**(C, F)**, red] was localized to a few cells within the PTS (*arrows*), separate from the OCT4^+^ subpopulation (**C, F**, green, *arrowheads*). All slides were counter-stained with 4’,6’-diamino-2-phenylindole. **A-C**: scale bars: 20 µm. **D, E, F**: magnified view of **A, B, C,** respectively.

IF IHC staining was performed on three representative LMCA samples from the original cohort of nine patients used for DAB IHC staining, to determine the expression of cathepsins B, D and G in relation to the CSC subpopulations, previously identified. Interestingly, cathepsin B was expressed by cells within the TNs (Figure 2A, red, *arrows*) and the OCT4^+^ cells within the PTS (Figure 2A, green, *arrowheads*). Similarly, cathepsin D was expressed by the OCT4^−^ cells within the TNs (Figure 2B, red, *arrows*) and the OCT4^+^ CSCs within the PTS (Figure 2B, green, *arrowheads*). This was in contrast to cathepsin G which was localized to a few cells within the PTS (Figure 2C, red, *arrows*), separate from the OCT4^+^ subpopulation (Figure 2C, green, *arrowheads*). Images of the individual stains are presented in Figure S2. Minimal staining was present on the negative control (Figure S2G), confirming the specificity of the primary antibodies used.

### Cell Counting and Statistical Analyses

**Figure 3:**
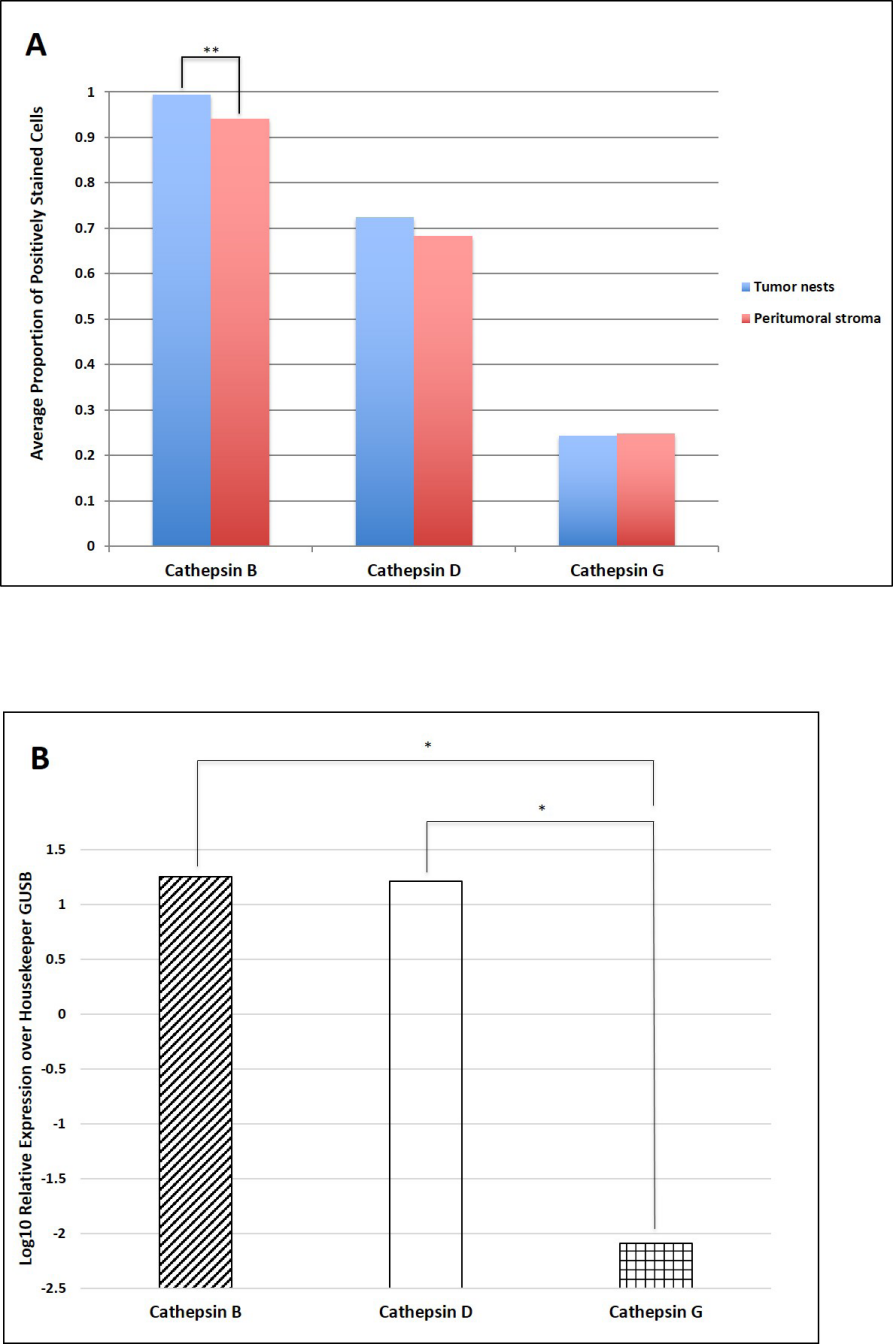
Cell counting of 3,3-diaminobenzidine immunohistochemical-stained slides of liver metastasis from colon adenocarcinoma demonstrating cells within the tumor nests (TNs) and peri-tumoral stroma (PTS) stained positively for cathepsins B, D and G. There was statistical difference in the abundance between the cells within the TNs and those within the PTS (**p < 0.001) **(A)**. Average relative expression of cathepsins B, D, and G mRNA transcripts in samples of colorectal adenocarcinoma metastasis to the liver from six patients, normalized over housekeeping gene GUSB and presented as relative units (*p < 0.05) **(B)**.

Cell counting on DAB IHC-stained slides for cathepsins B, D and G (Figure 3A) in the nine samples of LMCA demonstrated significantly greater expression of cathepsin B by the cells within the TNs compared to those within the PTS (*t* = 4.384, *p* = 0.007). There was no significant difference between cells stained positively within the TNs and those within the PTS for cathepsin D (*p* = 0.589) and cathepsin G (*p* = 0.927).

### NanoString mRNA Expression Analysis

NanoString mRNA expression analysis confirmed the presence of mRNA transcripts for cathepsin B, D and G relative to the housekeeping gene GUSB in all six snap-frozen LMCA samples (Figure 3B). Statistical analysis of the NanoString data demonstrated that there was no significant difference between the mean level of expression of cathepsin B compared to that of cathepsin D (*p* = 0.345). However, the mean level of expression of cathepsin B (*p* = 0.028) and the mean level of expression of cathepsin D (*p* = 0.028) were significant greater than that of cathepsin G.

### Western Blotting

**Figure 4:**
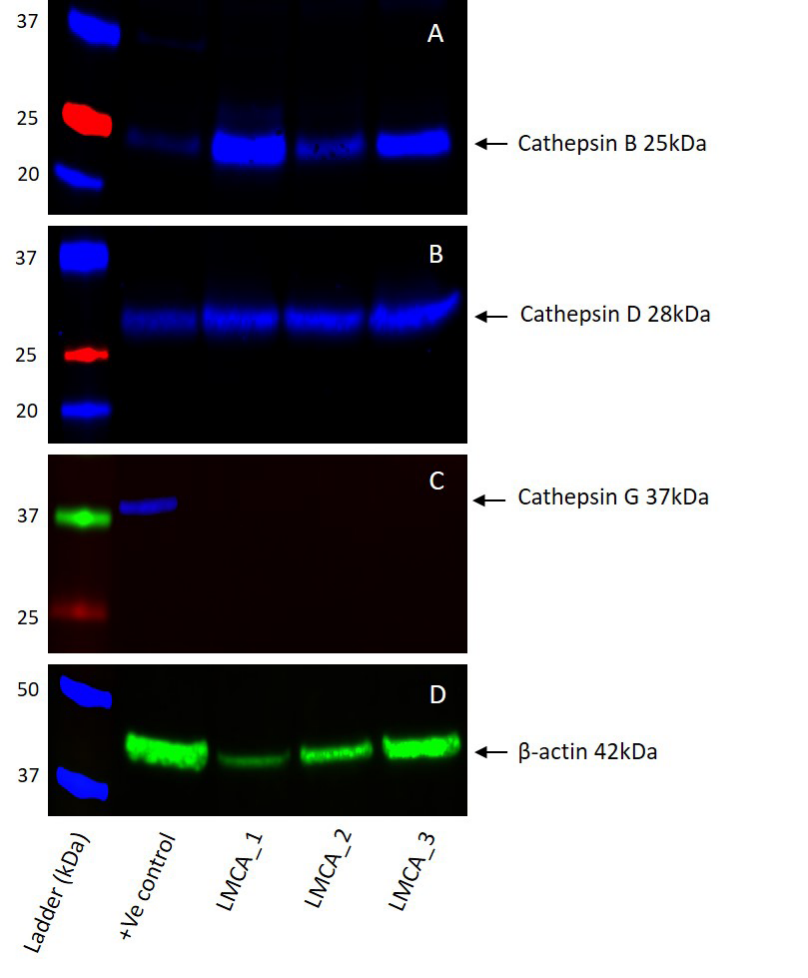
Representative Western blot images of total protein extracted from samples of liver metastasis from colon adenocarcinoma from three patients demonstrating the presence of cathepsin B **(A)** and cathepsin D **(B)**, but not cathepsin G **(C)**. β-actin was used as a housekeeping protein **(D)**.

WB of the three snap-frozen LMCA samples demonstrated the presence of bands at the expected molecular weight for cathepsin B (Figure 4A) and cathepsin D (Figure 4B). Cathepsin B was detected at the appropriate molecular weight of 25 kDa in all three LMCA samples. Cathepsin D was detected at the corresponding molecular weight of 28 kDa in all three LMCA samples. Cathepsin G was not detected in any of the LMCA tissue samples at the expected molecular weight of 29 kDa (Figure 4C), with specificity of the antibody confirmed in the positive control, which demonstrated detection of a band at the expected molecular weight of 37 kDa, consistent with the manufacturer’s product information. β-actin (Figure 4D) confirmed approximate equivalent protein loading for all LMCA samples examined. The tonsillar tissue confirmed specificity for cathepsin B and cathepsin D. The recombinant cathepsin G protein confirmed specificity for cathepsin G. The rabbit and mouse IgG isotype negative controls demonstrated minimal staining, as expected (Figure S3A,B ). The complete WB for cathepsins B, D and G are provided (Figure S3C-D).

### Enzymatic Activity Assays

**Figure 5:**
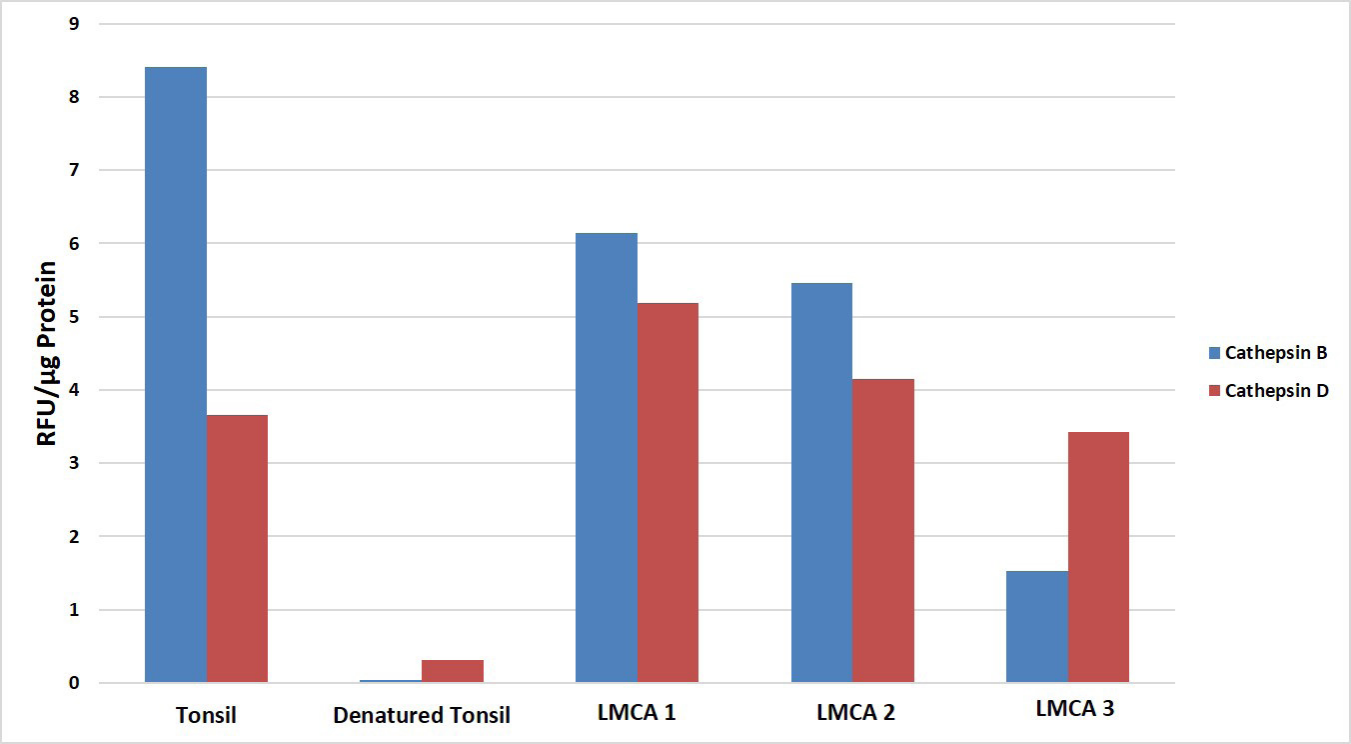
Enzymatic activity assays showed both cathepsin B and cathepsin D were active in all three samples of liver metastasis from colon adenocarcinoma relative to the positive and negative controls. Their activity is expressed as relative fluorescent unit (RFU).

To determine the functional activity of both cathepsin B and cathepsin D, which were detected by WB, we performed enzymatic activity analysis on the same three snap-frozen LMCA tissue samples, for both cathepsins. All the LMCA samples demonstrated enzymatic activity of cathepsin B and cathepsin D relative to that of the positive and negative control tissues (Figure 5). This provides evidence that these cathepsins are functional.

## Discussion

The expression of ESC markers in CRC has been well documented and various combinations of markers have been reported. OCT4, SOX2, NANOG, Krüppel-like factor 4 (KLF4) and c-MYC play a significant role in triggering pluripotency in somatic cells, and they have been used to identify CSC subpopulations within various types of cancer (31). CD44, LGR5 and EpCAM are markers that are of particular relevance to CRC (11). Furthermore, CD133^+^ cells that have been isolated from primary colon cancer and liver metastases, have been shown to be capable of initiating tumor growth in immunodeficient mice (32).

The self-renewing capacity of CSCs and their persistence despite conventional therapy offers a possible explanation for tumor recurrence and metastasis (33). CSCs are able to invade and metastasize by acquiring an epithelial-mesenchymal transition (EMT) phenotype, which can be determined by examining the expression of E-cadherin and vimentin (34). Several signaling pathways are known to promote stem cell maintenance and induce EMT (33). In particular, Wnt/β-catenin signaling has been reported to significantly influence the regulation of growth and maintenance of CRC CSCs (34). Similarly, the Notch signaling pathway is thought to contribute to colon CSC viability, tumorigenicity and self-renewal (33).

Our previous characterization of three putative CSC subpopulations within LMCA led us to propose the existence of a hierarchy of CSCs within this tumor (12). Interestingly, IF IHC staining showed the expression of cathepsin B and cathepsin D by the OCT4^+^ CSC subpopulation within the PTS of the LMCA samples we have examined. However, cathepsin G was localized to a separate OCT4^−^ subpopulation.

*In vitro* and *in vivo* studies have demonstrated the involvement of the RAS in proliferative signaling, evasion of growth suppressors, resistance of cell death, angiogenesis, reprogramming of energy metabolism, inflammation, cell migration, invasion and metastasis (35). The expression of components of the RAS by these CSCs, suggests the presence of a paracrine RAS involved in the regulation of CSCs (19). Furthermore, ACE inhibitors and ATIIR1 antagonists, which inhibit ATII production and action respectively, appear to exert inhibitory effects on cancer progression, vascularization and metastasis (36).

We have previously reported the expression of cathepsin B and cathepsin D by CSC subpopulations within oral tongue SCC (21) and IDHWGB (20), suggesting the presence of bypass loops for the RAS. To the best of our knowledge, this is the first report demonstrating the expression of cathepsin B and cathepsin D, and possibly cathepsin G in LMCA, suggesting the presence of RAS bypass loops.

DAB IHC staining and WB confirmed protein expression of cathepsin B and cathepsin D and the enzyme activity assays demonstrated the functional activity of these cathepsins. IF IHC staining showed the expression of cathepsin B and cathepsin D to the CSCs within the TNs and the PTS. Cathepsin G was localized to only a few cells within the PTS and it is not surprising that it was below detectable levels on WB. The expression of cathepsins B, D and G was confirmed at the transcriptional level with NanoString mRNA expression analysis. This indicates more abundant transcript expression of cathepsin B and cathepsin D, compare to that of cathepsin G, which was detected at low levels.

Cathepsin B can directly or indirectly degrade ECM in CRC by stimulating other proteases or by blocking their inhibitors. *In vivo* studies have demonstrated inhibition of cathepsin B by selective compounds leads to in reduced liver metastases by up to 60% and reduced liver metastases burden up to 80% (6).

Overexpression of cathepsin D has been associated with many types of cancers such as gastric carcinoma (37), melanoma (38), ovarian cancer (39) and breast cancer (40). It has been postulated that cathepsin D directly promotes tumor growth by degrading and remodeling the basement membrane and the stroma surrounding the tumor, and also acts indirectly by stimulating other enzymes or in conjunction with other cathepsins (2). Kirana et al (2). have demonstrated increased cathepsin D expression in cells from the main tumor body in late stage CRC to be significantly associated with subsequent distant metastasis and reduced cancer-specific survival.

Both cathepsin B and cathepsin D have been shown to be involved in ECM degradation in CRC, and levels of these cathepsins and their activity have been reported to be increased in the invasion edge of CRC (6). The expression of cathepsin B and cathepsin D by the CSC subpopulations within LMCA suggest these CSC subpopulations may be a therapeutic target. Further investigation into the expression of the RAS by these CSC subpopulations may underscore an effective targeted treatment strategy for this cancer.

This report presents novel finding of the expression of cathepsin B, cathepsin D, and possibly cathepsin G by the putative CSC subpopulations within LMCA.

## Limitations

1. Data from this study consists of a relatively small sample size, although it provides the foundation for future larger studies.
2. Further *in vitro* and *in vivo* data is required to validate the functional role of these cathepsins within LMCA.
3. Detection and functional analysis of components of the RAS in relation to CSCs in LMCA is required to underscore how these CSCs can be most effectively targeted.

## Ethics Statement

This study was carried out with the approval of the Central Health and Disability Ethics Committee (ref. no. 13/CEN/130) with written informed consent from all subjects in accordance with the Declaration of Helsinki.

## Author Contributions

TI and ST formulated the study hypothesis. TI, SW and ST designed the study. SW, SM, HB, ST and TI interpreted the DAB and IF IHC data. SW, ST and TI interpreted the NanoString mRNA data. SM performed cell counting on DAB IHC stained slides. BVS performed WB and enzymatic activity assays. RM conducted statistical analysis and interpreted the results. SM, ST and TI drafted the manuscript. All authors commented on and approved the manuscript.

## Conflict of Interest Statement

TI and STT are inventors of the PCT patent applications Cancer Diagnosis and Therapy (No. PCT/NZ2015/050108), and Cancer Therapeutic (PCT/NZ2018/050006). The authors declare that the research was conducted in the absence of any commercial or financial relationships that could be construed as a potential conflict of interest. 

## Supplementary material

Figure S1 3,3-Diaminobenzidine (DAB) immunohistochemical (IHC) staining of normal liver adjacent to liver metastasis from colon adenocarcinoma (LMCA) showing minimal staining of hepatocytes for cathepsin B (A, brown), cathepsin D (B, brown) and cathepsin G (C, brown). Positive controls of DAB IHC staining for cathepsin B (D, brown), cathepsin D (E, brown) and cathepsin G (F, brown) demonstrating the expected staining patterns in human placenta, breast cancer and bone marrow, respectively. The negative control (G) of a LMCA section demonstrated no staining. Nuclei were counter-stained with hematoxylin (A-G, blue). Original magnification: 400x.

Figure S2 Split images of immunofluorescence immunohistochemical-stained sections of colon adenocarcinoma metastasis to the liver (LMCA) shown in Figure 2 demonstrating co-expression of cathepsin B (A, red) with OCT4 (B, green), cathepsin D (C, red) and OCT4 (D, green), cathepsin G (E, red) and OCT4 (F, green). A negative control (G) to test the specificity of the fluorescent secondary antibodies is performed on a section of LMCA. Cell nuclei were counter-stained with 4′,6-diamidino-2-phenylindole (A-G, blue). Scale bars: 20 µm.

Figure S3 Western blots of matched IgG isotype negative controls with a fluorescent secondary antibody confirming specificity for the cathepsin B and cathepsin D bands with β-actin as a housekeeping protein (A), and with a HRP-conjugated secondary confirming non-specific binding for the 50 kDa bands present in the cathepsin G blot (B). Complete western blot images for cathepsin B (blue) and β-actin (green) (C), cathepsin D (D) and cathepsin G (E).
