# Supplementary figures and images for: Expression and Localization of Cathepsins B, D and G in Cancer Stem Cells in Liver Metastasis From Colon Adenocarcinoma
Source: Front Surg. 2018 Jun 7;5:40. doi: 10.3389/fsurg.2018.00040 (PMC6110174; doi:10.3389/fsurg.2018.00040)

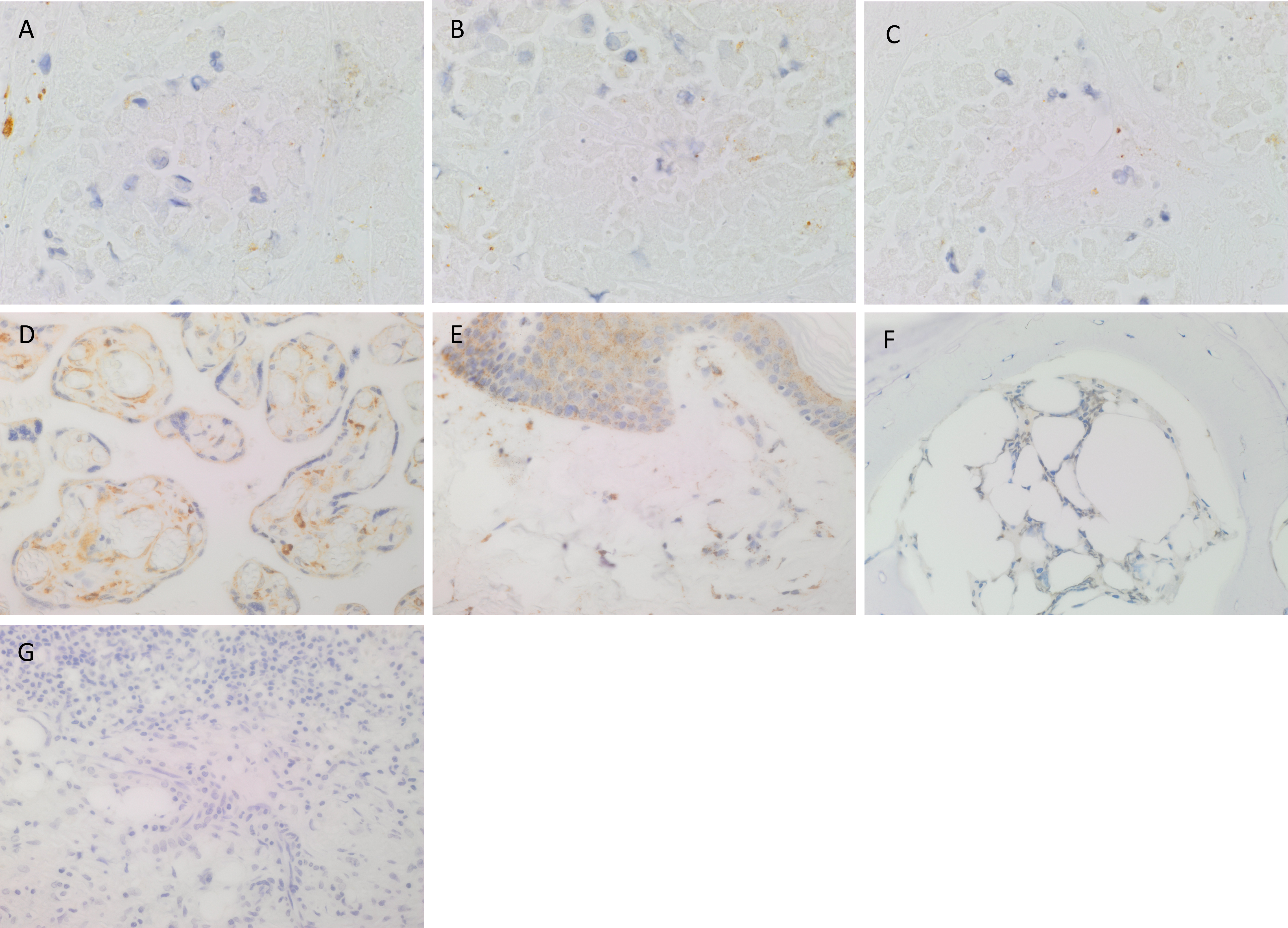

Supplement: Figure S1 — 3,3-Diaminobenzidine (DAB) immunohistochemical (IHC) staining of normal liver adjacent to liver metastasis from colon adenocarcinoma (LMCA) showing minimal staining of hepatocytes for cathepsin B (A, brown), cathepsin D (B, brown) and cathepsin G (C, brown). Positive controls of DAB IHC staining for cathepsin B (D, brown), cathepsin D (E, brown) and cathepsin G (F, brown) demonstrating the expected staining patterns in human placenta, breast cancer and bone marrow, respectively. The negative control (G) of a LMCA section demonstrated no staining. Nuclei were counter-stained with hematoxylin (A-G, blue). Original magnification: 400x. [file Image1.TIF]

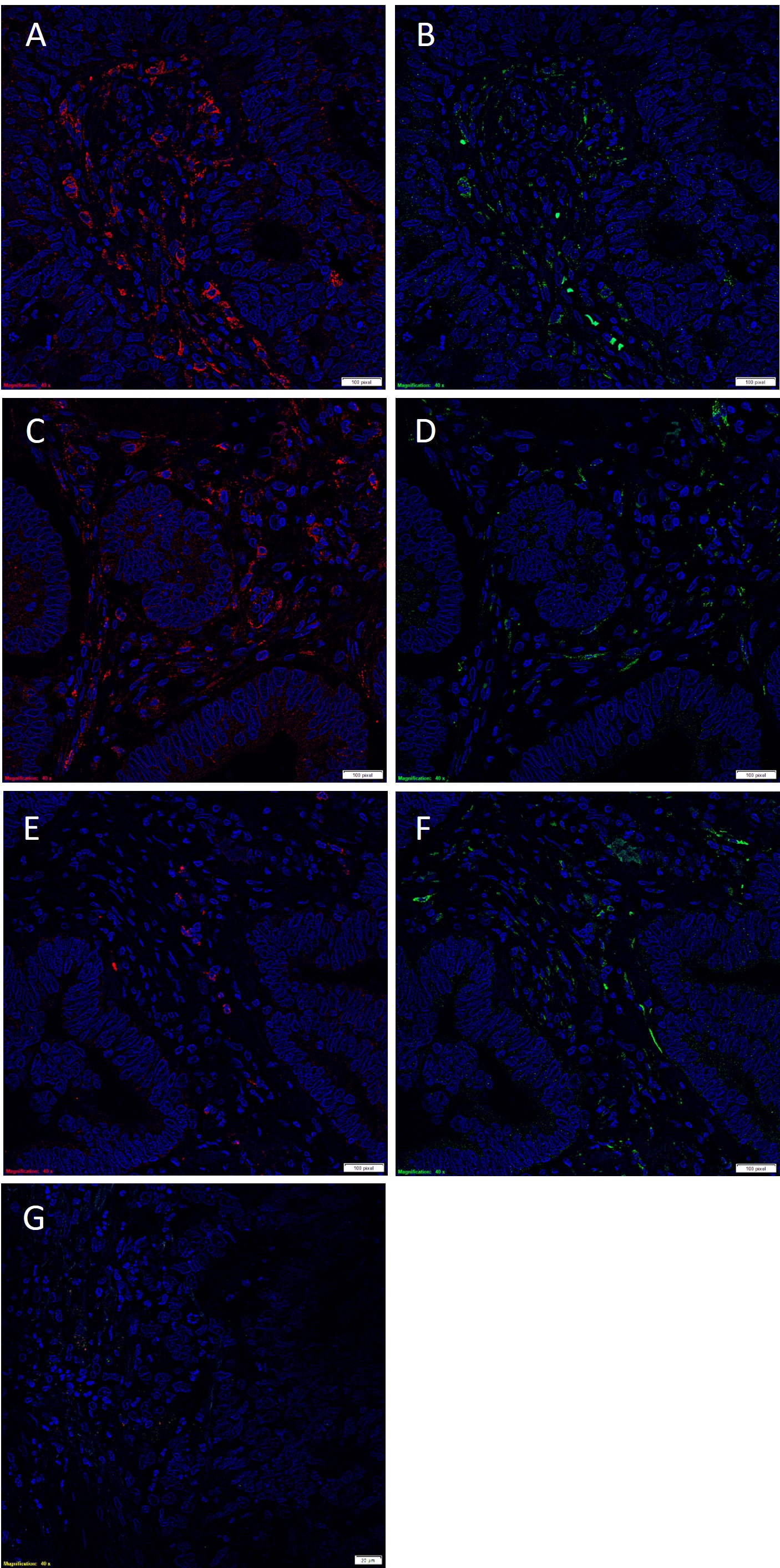

Supplement: Figure S2 — Split images of immunofluorescence immunohistochemical-stained sections of colon adenocarcinoma metastasis to the liver (LMCA) shown in Figure 2 demonstrating co-expression of cathepsin B (A, red) with OCT4 (B, green), cathepsin D (C, red) and OCT4 (D, green), cathepsin G (E, red) and OCT4 (F, green). A negative control (G) to test the specificity of the fluorescent secondary antibodies is performed on a section of LMCA. Cell nuclei were counter-stained with 4′,6-diamidino-2-phenylindole (A-G, blue). Scale bars: 20 µm. [file Image2.JPEG]

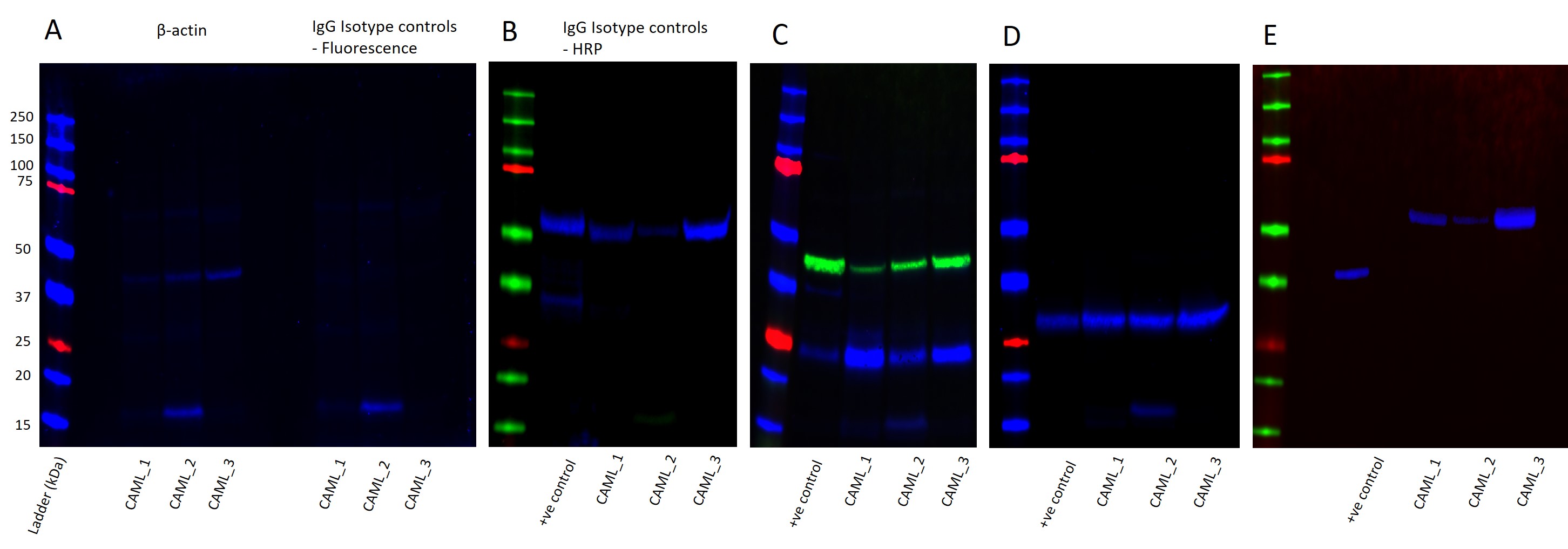

Supplement: Figure S3 — Western blots of matched IgG isotype negative controls with a fluorescent secondary antibody confirming specificity for the cathepsin B and cathepsin D bands with β-actin as a housekeeping protein (A), and with a HRP-conjugated secondary confirming non-specific binding for the 50 kDa bands present in the cathepsin G blot (B). Complete western blot images for cathepsin B (blue) and β-actin (green) (C), cathepsin D (D) and cathepsin G (E). [file Image3.JPEG]
